# Systematische und qualitätsgesicherte Früherkennung des sporadischen Mammakarzinoms: Update Screening-Effekte und wissenschaftliche Studien

**DOI:** 10.1007/s00117-020-00803-1

**Published:** 2021-01-25

**Authors:** Walter Heindel, Karin Bock, Gerold Hecht, Sylvia Heywang-Köbrunner, Vanessa Kääb-Sanyal, Katja Siegmann-Luz, Stefanie Weigel

**Affiliations:** 1 Klinik für Radiologie und Referenzzentrum Mammographie Münster, Universität Münster (WWU) und Universitätsklinikum Münster (UKM), Albert-Schweitzer-Campus 1, Gebäude A1, 48149 Münster, Deutschland; 2 Referenzzentrum Mammographie Südwest, Bahnhofstraße 7, 35037 Marburg, Deutschland; 3 Referenzzentrum Mammographie Nord, Heiligengeiststraße 28, 26121 Oldenburg, Deutschland; 4 Referenzzentrum Mammographie München, Sonnenstraße 29, 80331 München, Deutschland; 5 Geschäftsstelle der Kooperationsgemeinschaft Mammographie, Goethestraße 85, 10623 Berlin, Deutschland; 6 Referenzzentrum Mammographie Berlin, Straße des 17. Juni 106–108, 10623 Berlin, Deutschland

**Keywords:** Brustkrebs, Qualitätsgesichertes Mammographie-Screening-Programm, Krebsregister, Fortgeschrittene Tumorstadien, Brustkrebsspezifische Mortalität, Breast cancer, Quality-assured mammography screening program, Cancer registry, Advanced tumor stages, Breast cancer-related mortality

## Abstract

**Hintergrund:**

Allen Frauen in Deutschland im Alter von 50 bis 69 Jahren wird seit 2009 flächendeckend ein qualitätsgesichertes Mammographie-Screening-Programm angeboten, das an die Europäischen Leitlinien angelehnt ist. Dieser Übersichtsartikel fasst den aktuellen Stand wissenschaftlicher Bewertungen dieses nationalen Brustkrebs-Früherkennungsprogramms zusammen und gibt einen Ausblick auf laufende Studien zur Effektivitätsprüfung und Weiterentwicklung.

**Ergebnisse:**

Aufgrund der erreichten Diagnosevorverlagerung mit Abnahme fortgeschrittener Brustkrebserkrankungen nach wiederholter Screening-Teilnahme ist ein Rückgang der brustkrebsspezifischen Mortalität zu erwarten; das Ausmaß wird die laufende ZEBra-Studie zur Mortalitätsevaluation zeigen.

**Schlussfolgerung:**

Auf vier Feldern zeichnet sich Potenzial zur weiteren Effektivitätssteigerung der Brustkrebs-Früherkennung ab: 1. Frauen sollten das Früherkennungsangebot der Krankenkassen häufiger wahrnehmen; bisher nimmt durchschnittlich nur etwa jede zweite anspruchsberechtigte Frau zwischen 50 und 69 Jahren am Screening-Programm teil. 2. Erweiterung der Anspruchsberechtigung über das 70. Lebensjahr hinaus. 3. Die Weiterentwicklung der digitalen Mammographie zur digitalen Brust-Tomosynthese verspricht die Zahl falsch-positiver Rückrufe zu reduzieren bei gleichzeitiger Sensitivitätssteigerung. 4. Für die wenigen Frauen in der anspruchsberechtigen Gruppe mit extrem dichter Brust sind erweiterte Screening-Strategien wissenschaftlich zu überprüfen.

## Abstract (en)

**Background:**

A quality-assured mammography screening programme has been available since 2009, nationwide, to all women in Germany between the ages of 50 and 69. The programme is based on the European Guidelines. In this review article the authors summarize the current status of scientific assessments of this national early detection programme for breast cancer and provide an outlook regarding ongoing studies on effectiveness tests and further development.

**Results:**

We expect a decline in mortality rates relating to breast cancer as a result of successfully bringing diagnoses forward and a decrease in advanced breast cancer after a repeated screening. The extent will be shown in the current ZEBra study on mortality evaluation.

**Conclusion:**

Potential for a further increase in the effectiveness of the systematic early detection of breast cancer can be identified in four areas: (1) More women should take advantage of the early detection opportunities offered by the medical insurance funds; so far, on average, only about 50% of the women between 50 and 69 who are entitled to a screening examination actually take part in the programme. (2) Entitlement to take part in the programme should be extended to women over 70. (3) The further development of digital mammography towards digital breast tomosynthesis promises to reduce the number of false positive recalls while at the same time increasing sensitivity. (4) There should be scientific studies relating to an extension of screening strategies for the small number of women in the entitlement range who have extremly dense breasts.

Brustkrebs ist in Deutschland – wie in den meisten anderen westlichen Staaten – mit Abstand die häufigste Tumorerkrankung bei Frauen. Gleichzeitig stellt Brustkrebs für Frauen das größte Risiko eines vorzeitigen Todes durch eine Tumorerkrankung dar. Nach den neuesten Angaben des Robert Koch-Instituts (RKI) erkranken in Deutschland jährlich mehr als 74.950 Frauen neu an einem invasiven oder einem In-situ-Mammakarzinom; 18.570 Frauen versterben in unserem Land jedes Jahr durch Brustkrebs [1].

Eine systematische Brustkrebs-Früherkennung soll die Sterblichkeit an Brustkrebs senken:

- Für Frauen mit familiärer Belastung für Brust- und Eierstockkrebs wurde die sog. *intensivierte Früherkennung* aufgebaut; 23 universitäre Zentren bieten im interdisziplinären Verbund von Humangenetik, Gynäkologie und Radiologie eine Risikokalkulation und ggf. Genanalyse, eine standardisierte Beratung sowie die Durchführung primärer, sekundärer und tertiärer präventiver Maßnahmen an [2].
- Für alle Frauen im Alter von 50 bis 69 Jahren – nicht genetisch bedingter, sog. sporadischer Brustkrebs ist in der Regel eine Erkrankung des höheren Lebensalters – wurde nach einem einstimmigen Beschluss des Deutschen Bundestags in den Jahren 2005 bis 2009 flächendeckend ein *Mammographie-Screening-Programm*, angelehnt an die Europäischen Leitlinien, eingeführt [3]. Als Besonderheit werden alle anspruchsberechtigten Frauen jedes zweite Jahr schriftlich zu einer Mammographie-Untersuchung in eine sog. Screening-Einheit eingeladen. Die Screening-Mammographie wird dort nur von speziell geschulten Fachkräften an arbeitstäglich streng kontrollierten, modernen Geräten durchgeführt. Das Bundesamt für Strahlenschutz (BfS) hat 2018 dazu eine neue Nutzen-Risiko-Bewertung veröffentlicht [4]. Krebs-Früherkennungsprogramme stellen besondere Anforderungen an die Qualitätssicherung und das Qualitätsmanagement, die alle Schritte der Screening-Kette erfassen und einen ständigen Verbesserungsprozess implizieren (*lernendes System*; [5]).

Nach mehr als 10 Jahren Regelversorgung durch das Mammographie-Screening-Programm konzentriert sich die vorliegende Publikation auf Effektanalysen und fasst Konzepte zur wissenschaftlich fundierten Weiterentwicklung zusammen.

## Struktur und Versorgungskette des qualitätsgesicherten Programms

Das deutsche Mammographie-Screening-Programm wurde durch die Kassenärztliche Bundesvereinigung (KBV) und die damaligen Spitzenverbände der Krankenkassen, heute GKV-Spitzenverband, als bevölkerungsbezogenes und qualitätsgesichertes Brustkrebs-Früherkennungsprogramm eingeführt. Organisation, Qualitätssicherung und Evaluation der Versorgungskette und aller daran beteiligten Personen sind auf Basis der Europäischen Leitlinien in der Krebsfrüherkennungs-Richtlinie (KFE-RL) und der Anlage 9.2 des Bundesmantelvertrags für Ärzte (BMV-Ä) verbindlich vorgegeben. Zur bundesweiten Organisation und Koordination des Programms wurde die Kooperationsgemeinschaft Mammographie (KoopG) bestehend aus einer Geschäftsstelle in Berlin sowie fünf eigenständigen, regional verantwortlichen Referenzzentren gegründet.

Zur Sicherstellung der Qualität erfolgt die Früherkennung im Rahmen des Mammographie-Screening-Programms ausschließlich in von der KoopG zertifizierten Screening-Einheiten. Jede Screening-Einheit ist einem der fünf Referenzzentren zugeordnet. Die Referenzzentren sind dabei u. a. für die externe Überwachung der Qualitätssicherung und die Fortbildung, Betreuung sowie Beratung der am Programm teilnehmenden Ärzte und radiologischen Fachkräfte verantwortlich. 2017 wurden die Referenzzentren ihrerseits durch die unabhängige EUREF („European Reference Organisation for Quality Assured Breast Cancer Screening and Diagnostic Services“) als „European Reference Centres for Breast Screening“ zertifiziert.

**Figure 1:**
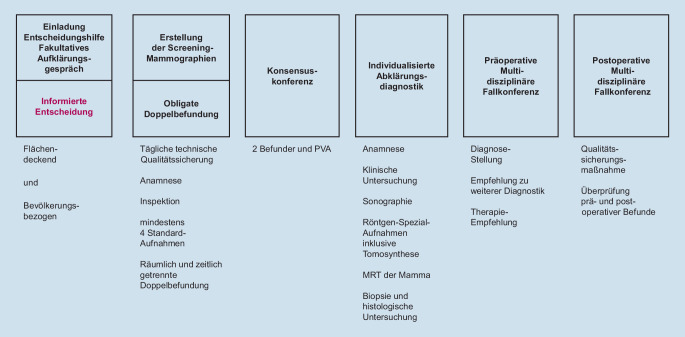

Die Versorgungskette im Mammographie-Screening-Programm (MSP) ist in Abb. 1 dargestellt. Auf Basis der regelmäßigen Auswertungen der Qualitätssicherungsmaßnahmen, der Ergebnisse des MSP sowie Änderungen rechtlicher Rahmenbedingungen und Weiterentwicklungen in Medizin und Technik werden die Vorgaben des MSP regelmäßig aktualisiert.

Eine wichtige Anpassung ist die konsequente Stärkung der informierten Entscheidung der anspruchsberechtigten Frauen. Der Einladung zur Früherkennungsuntersuchung an die Frau liegt hierzu eine vom unabhängigen Institut für Qualität und Wirtschaftlichkeit im Gesundheitswesen (IQWiG) entwickelte, evidenzbasierte Entscheidungshilfe bei. Grundsätzlich wird den Frauen zudem vor der Teilnahme ein persönliches Aufklärungsgespräch mit einem Screening-Arzt angeboten. Ergänzende und weitergehende Informationen werden von der KoopG im Internet [3] sowie auf den *Social-Media*-Kanälen Facebook und Instagram bereitgestellt [6].

Eine weitere Besonderheit des organisierten Früherkennungsprogramms ist die jährliche Qualitätsdarlegung und Ergebnisauswertung zum Nachweis der Effektivität des Programms anhand standardisierter Indikatoren aus den Europäischen Leitlinien. Die zuletzt veröffentlichten Jahresberichte Evaluation und Qualitätssicherung 2018 belegen eindrücklich die effektive Früherkennung im MSP [7].

## Aktuelle Performance-Parameter

**Figure 2:**
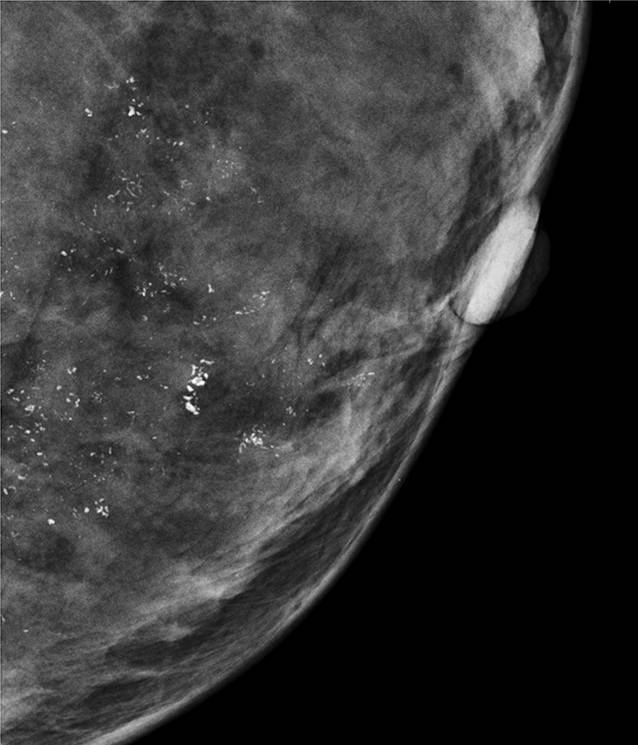

Basierend auf einer Einladung alle 2 Jahre, erhält jeweils die Hälfte der anspruchsberechtigten Frauen pro Jahr eine Einladung zur Mammographie. Im Jahr 2018 waren dies 5,7 Mio. Einladungen; rund 50 % der Frauen nahmen die Einladung an. Bei den 2,9 Mio. untersuchten Frauen im Jahr 2018 wurden über 17.000 Mammakarzinome entdeckt, 20 % davon als In-situ-Karzinom (Abb. 2).

**Figure 3:**
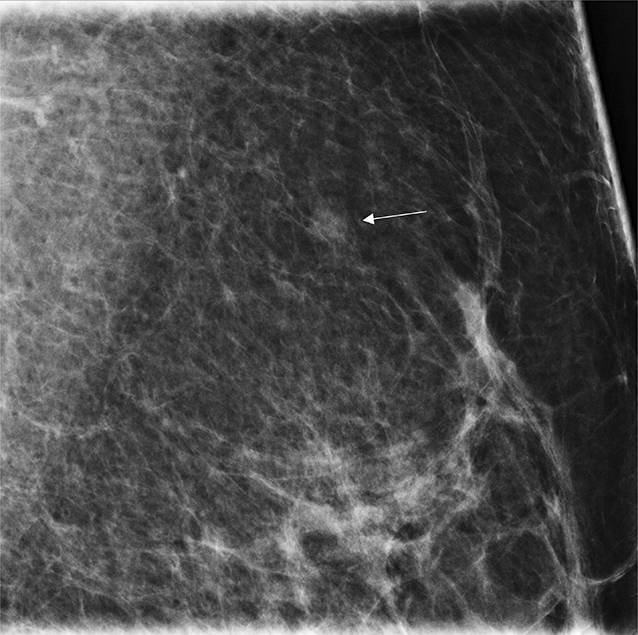

Die im Folgerunden-Screening entdeckten invasiven Karzinome sind überwiegend klein und ohne Lymphknotenbefall: 34 % sind ≤1 cm (Abb. 3), 80 % sind maximal 2 cm groß (pT1) und bei 82 % der im Screening-Programm diagnostizierten invasiven Karzinome sind die axillären Lymphknoten frei von Metastasen.

Lediglich 21 % der im Screening detektierten Karzinome sind den prognostisch ungünstigen Stadien UICC II+ zuzuordnen; vor Einführung des Screenings waren dies noch 56 % der entdeckten Karzinome. Bis auf die Teilnahmerate erfüllen alle Leistungsparameter die empfohlenen Referenzwerte der Europäischen Leitlinien. Würden noch mehr Frauen das Angebot des qualitätsgesicherten Screening-Programms wahrnehmen, ließe sich gesundheitspolitisch der Früherkennungseffekt noch weiter steigern [8].

Die straffe Organisation der Versorgungskette soll dafür sorgen, dass die Teilnehmerinnen nicht unnötig durch Wartezeiten und weitere Untersuchungen belastet werden. 97 % der Frauen erhalten ihr Screening-Ergebnis innerhalb von 7 Werktagen. Bei wiederholter Screening-Teilnahme sind lediglich bei 2,9 % der Frauen weitere Untersuchungen zur Abklärung von Auffälligkeiten erforderlich, die Frauen erhalten hierzu innerhalb einer Woche einen Termin. Bei nur 1,1 % aller im MSP untersuchten Frauen erfolgt eine Gewebeentnahme, wobei sich in über der Hälfte der Fälle (54 %) der Verdacht auf Brustkrebs bestätigt [7].

## Screening-Effekte

**Figure 4:**
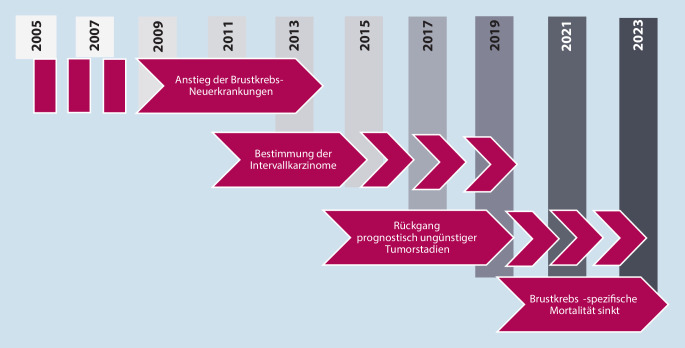

Der wissenschaftliche Beirat der KoopG hat ein Phasenmodell entwickelt, das die Effekte des MSP darstellt und vor allem die Analyse ihrer Wirksamkeit erlaubt (Abb. 4):

### Phase 1: Anstieg der Brustkrebs-Neuerkrankungen

Bei einem effektiven, bevölkerungsbezogenen Früherkennungsprogramm führt die präklinische Vorverlegung der Diagnosen zu einer Inzidenzsteigerung insbesondere früher Karzinomstadien.

**Figure 5:**
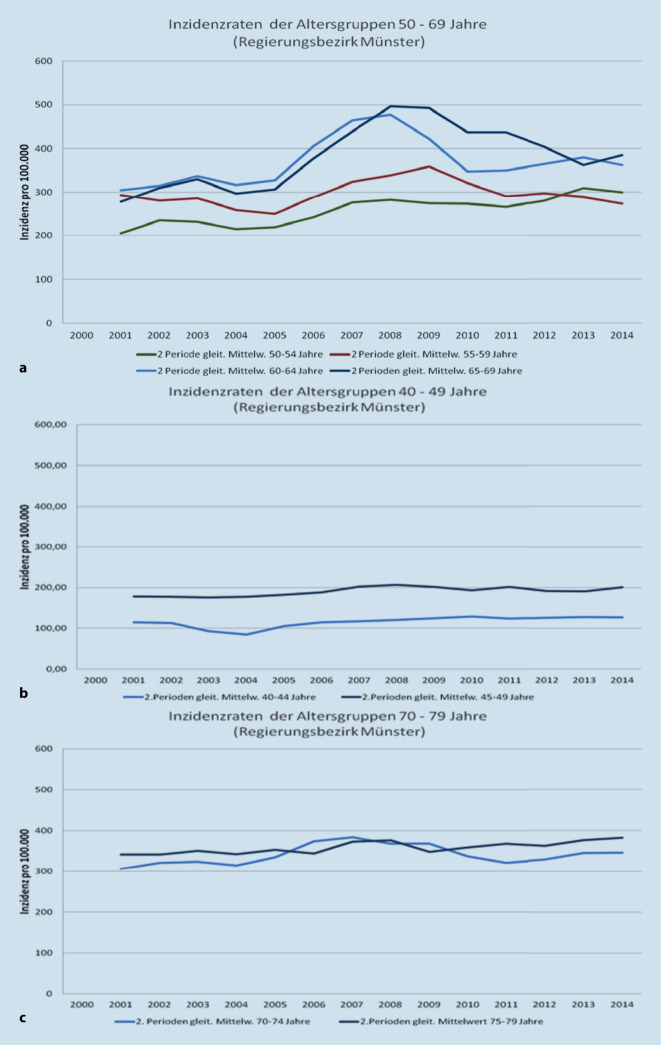

In Deutschland konnte dieser epidemiologische Effekt (Abb. 5) erstmals in der Region Münster beobachtet werden [9] und später entsprechend der bundesweiten Etablierung der systematischen Brustkrebs-Früherkennung in West-Deutschland eher als in Ost-Deutschland.

Dieser zu erwartende Effekt durch das flächendeckende Mammographie-Screening-Programm wurden in der Folge durch das RKI [1] genauso wie die aktuelle Analyse der Arbeitsgruppe um Katalinic et al. [10] bestätigt.

### Phase 2: Bestimmung der Intervallkarzinome

Intervallkarzinome sind Mammakarzinome, die zwischen 2 Screeningrunden, d. h. im Zeitraum von 24 Monaten nach einer unauffälligen Screening-Untersuchung (ggf. inklusive Abklärungsdiagnostik) außerhalb des Brustkrebs-Früherkennungsprogramms diagnostiziert werden. Dazu zählen sowohl invasive Mammakarzinome (ICD-10 C50) als auch In-situ-Karzinome (ICD-10 D05).

**Figure 6:**
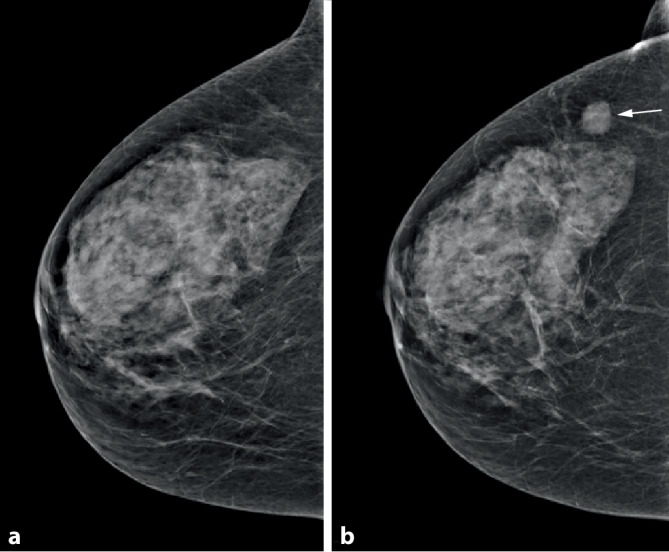

Intervallkarzinome sind in einem Screening-Programm unvermeidlich, da naturgemäß zu jedem Zeitpunkt neue Karzinome entstehen können. Bei einer radiologischen Kategorisierung stellen die allermeisten Intervallkarzinome sog. *echte Intervallkarzinome* dar [8], die zwischen den Screeningrunden, d. h. im Intervall, erstmals mammographisch abgrenzbar sind. In Abb. 6 wird diese häufigste Konstellation verdeutlicht.

Die Erhebung von Intervallkarzinomen erfordert einen Datenabgleich zwischen den Screening-Einheiten und einem Krebsregister. Das Landeskrebsregister Nordrhein-Westfalen (LKR NRW) hat diesen Datenabgleich in einer speziellen pseudonymisierten Form realisiert. Das LKR NRW spiegelt dabei die Intervallkarzinom-Verdachtsfälle über das Referenzzentrum Münster an die einzelnen Screening-Einheiten in NRW zurück. Diese validieren die Verdachtsfälle anhand ihrer Screening-Dokumentation. Der Anteil bestätigter Verdachtsfälle in NRW lag für das Screening-Jahr 2012 bei 95 %. Der Datenabgleich zur Identifikation von Intervallkarzinomen im LKR NRW erweist sich demnach als verlässlich.

In Niedersachsen wurde der Abgleich der pseudonymisierten Daten aller Screening-Teilnehmerinnen mit dem Epidemiologischen Krebsregister Niedersachsen (EKN) während des Modellprojekts Mammographie-Screening Weser-Ems (2002–2005) in Forschungsprojekten erprobt und findet inzwischen routinemäßig für ganz Niedersachsen statt. In Niedersachsen übermittelt das EKN die Intervallkarzinom-Verdachtsfälle analog über das Referenzzentrum Nord an die jeweiligen Screening-Einheiten. Anhand der Screening-Dokumentation findet eine Validierung der Verdachtsfälle in der Screening-Einheit und im Referenzzentrum und abschließend eine Rückinformation an das EKN (Intervallkarzinom ja/nein) statt. Zusätzlich fordern die niedersächsischen Screening-Einheiten von den Ärztinnen und Ärzten, die das Intervallkarzinom diagnostiziert haben, die pseudonymisierten Befundunterlagen für die einzelfallbezogene Qualitätssicherung an. Ziel ist die Kategorisierung der Intervallkarzinome. Hiermit soll eine fortwährende Qualitätsoptimierung des Screening-Programms gewährleistet werden.

Im Screening-Jahr 2012 lag sowohl in NRW wie in Niedersachsen die relative Intervallkarzinom-Rate im ersten Jahr nach der Screening-Untersuchung bei 22 % und im zweiten Jahr bei 45 % bzw. 46 % der regionalen Hintergrundinzidenz [11, 12]. Die Zielwerte der EU-Leitlinien werden damit in beiden Bundesländern erreicht.

### Phase 3: Abnahme fortgeschrittener Tumorstadien

Die Abnahme der Inzidenz fortgeschrittener Brustkrebsstadien ist der informativste Surrogatparameter für den Rückgang der Brustkrebssterblichkeit [13, 14].

Fortgeschrittene Brustkrebserkrankungen fassen das UICC-Stadium II oder höher zusammen (UICC II+). UICC II+ werden Mammakarzinome mit einem T1 N+-Stadium (ohne T1 N1mic) oder T2-Stadium bzw. höher zugeordnet.

Das Landeskrebsregister NRW berichtete über den Verlauf der Inzidenzen fortgeschrittener Mammakarzinome (UICC II+) unter regulärer Teilnahme für zwei aufeinander folgende 24-Monats-Zeiträume [14]. Die Inzidenz bestand jeweils aus der Summe fortgeschrittener Mammakarzinome pro 100.000 Teilnehmerinnen, detektiert im Screening und im vorhergehenden 24-monatigen Intervall.

Die altersstandardisierte Inzidenzrate fortgeschrittener Mammakarzinome lag für den Zeitraum von 24 Monaten nach dem ersten Screening bei 291,6 pro 100.000 Frauen und für den Zeitraum von 24 Monaten nach dem ersten nachfolgenden Screening bei 275,0/100.000. Verglichen mit der 2‑Jahres-Inzidenz von 349,4/100.000 vor Beginn des Mammographie-Screening-Programms entsprach dies einer relativen Reduktion der Inzidenz bei regulärer Teilnahme um 16,5 % bzw. 21,3 %. In einer Metaanalyse randomisierter Screening-Studien war eine Reduktion der Inzidenz fortgeschrittener Mammakarzinome um 20 % mit einer 28 %igen Senkung der Brustkrebssterblichkeit verbunden [15].

Diese aktuellen Ergebnisse machen wie die Analysen des RKI [1] eine Senkung der Brustkrebsmortalität durch regelmäßige Screening-Teilnahme wahrscheinlich.

### Phase 4: Senkung der Brustkrebsmortalität

Die Brustkrebsmortalität sinkt seit den 2000er Jahren kontinuierlich. Katalinic et al. konnten zeigen, dass die Brustkrebsmortalität der Screening-Anspruchsberechtigten in den Altersklassen 50–59 Jahre und 60–69 Jahre nach Screening-Einführung signifikant gesunken ist [10]. Die Autoren führen dies auf die Effekte des MSP zurück.

Zur Evaluation des Einflusses des deutschen MSP auf die Mortalität ist eine Zusammenführung von Daten aus verschiedenen Quellen notwendig. Deshalb wurden der sog. ZEBra-Studie im Auftrag des Bundesamts für Strahlenschutz von 2012 bis 2017 zwei Machbarkeitsstudien vorgeschaltet. Unter Federführung des Instituts für Epidemiologie und Sozialmedizin der Universität Münster wurden datenschutzkonforme und praktikable Konzepte für die Datenzusammenführung beispielsweise von Krebsregistern und Krankenkassen entwickelt. Förderer sind das Bundesministerium für Umwelt, Naturschutz und nukleare Sicherheit (BMU), das Bundesministerium für Gesundheit (BMG), und die KoopG. Forschungspartner sind u. a. die Arbeitsgemeinschaft Deutscher Tumorzentren e. V., das Forschungszentrum Ungleichheit und Sozialpolitik der Universität Bremen – SOCIUM, das Leibniz-Institut für Präventionsforschung und Epidemiologie – BIPS GmbH und das LKR NRW. Ergebnisse sind im Jahr 2023 zu erwarten.

## Evidenzbasierte Weiterentwicklungen des MSP

Mögliche Weiterentwicklungen betreffen vor allem das Alter der anspruchsberechtigten Frauen und das Screening-Verfahren inklusive alternativer oder ergänzend eingesetzter bildgebender Methoden.

Vermutlich profitieren auch ältere Frauen bis 75 Jahre bei einer Lebenserwartung von mehr als 10 Jahren von einer regelmäßigen Screening-Mammographie. Einige Länder wie die Niederlande führen daher das Mammographie-Screening bis zu diesem Alter fort. In nationalen Leitlinien und Empfehlungen ist ebenfalls eine 2‑jährliche Screening-Mammographie in dieser Altersgruppe empfohlen [16, 17]. Das BfS hat vorgeprüft, welche Röntgenverfahren als Früherkennungsuntersuchung geeignet wären und das Mammographie-Screening zur Brustkrebs-Früherkennung für Frauen zwischen 70 und 74 Jahren mit oberster Priorität ausgewählt. Das BMU hat dieser Auswahl und Priorisierung zugestimmt und das BfS mit der ausführlichen Begutachtung beauftragt. Im Petitionsausschuss des Bundestages wurde die Anhebung der Altersgrenze für das Mammographie-Screening über das Alter von 70 Jahren hinaus am 26.10.2020 diskutiert und findet die Unterstützung des BMG [18].

Bei methodischen Forschungsansätzen zu alternativen oder ergänzenden Screening-Verfahren ist insbesondere das mögliche Nebenwirkungsspektrum zu beachten. Dazu gehören vor allem die Raten falsch-positiver Biopsien, mögliche Überdiagnosen, potenzielle Nebenwirkungen sowie strukturelle und finanzielle Machbarkeit. Nebenwirkungen sind für begrenzte Bevölkerungsgruppen dann vertretbar, wenn deren Erkrankungsrisiko und der erwartete Nutzen die potenziellen Nebenwirkungen rechtfertigt.

Als Selektionskriterium erscheint die mammographische Dichte von Bedeutung. Weigel et al. [19] konnten zeigen, dass die Programmsensitivität für das 2‑jährliche Mammographie-Screening-Programm durchschnittlich für alle Brustdichtegruppen ACR 1–4 bei 79,9 % liegt. Extrem dichte Drüsenkörper der Kategorie ACR 4 waren selten vertreten (<7 %), wiesen jedoch eine signifikant reduzierte Programmsensitivität von 50 % auf. Das heißt, innerhalb eines 2‑Jahres-Intervalls wurde die Hälfte der diagnostizierten Karzinome im Screening-Programm und die andere Hälfte außerhalb des Screening-Programms vor der nächsten Screening-Einladung als Intervallkarzinom diagnostiziert (Abb. 6). Die meisten Intervallkarzinome waren mammographisch neu aufgetretene Karzinome [19].

In einer neueren Arbeit konnten Weigel et al. zeigen, dass nicht alle Frauen mit sehr dichtem Drüsengewebe ein erhöhtes Brustkrebsrisiko haben. Die Kombination von Alter und Dichte verbessert die Abschätzung des Brustkrebsrisikos [20]. Mit steigender 5‑Jahres-Altersgruppe stieg die 2‑Jahres-Brustkrebsinzidenz von 5,0 ‰, 6,7 ‰, 8,5 ‰ auf 9,7 ‰ und unterschied sich unter den 55- bis 59-, 60- bis 64- und 65- bis 69-jährigen Frauen signifikant von der jüngsten Referenzgruppe 50–54 Jahre (Odds-Ratio [OR]: 1,34, 1,68 bzw. 1,93; *p*-Wert < 0,0001).

Innerhalb der Hauptkategorien ACR 2 und ACR 3 (fast 90 % aller gescreenten Frauen) steigen die Inzidenzen mit zunehmendem Alter bis zu einer Verdoppelung. Eine konsistent niedrige Inzidenz findet sich unabhängig von der Brustdichte bei jungem Screening-Alter und bei allen Frauen mit geringster Brustdichte.

### Screening mit digitaler Brust-Tomosynthese

Die Kombination von digitaler Brust-Tomosynthese und „full-field digital mammography“ (DBT + FFDM) führt zu einer Verbesserung der diagnostischen Genauigkeit im Vergleich zum alleinigen Einsatz der FFDM [21, 22]. Zur Vermeidung einer doppelten Strahlenexposition untersuchten neuere Studien die Performance der DBT mit synthetischer Mammographie (SM), eine FFDM-ähnliche Darstellung der Brust, rekonstruiert aus dem Datensatz der DBT. Prospektive Studien belegten, dass das Konzept DBT + SM dem Vorgehen DBT + FFDM nicht unterlegen ist [23–25]. Eine aktuelle Metaanalyse bewertet auf der Basis einer moderaten Evidenz, dass mittels DBT + SM die Detektionsrate invasiver Mammakarzinome um den Faktor 2 im Vergleich zur digitalen Mammographie gesteigert werden kann [26].

Insbesondere aufgrund von zu geringen Teilnehmerzahlen der ersten prospektiven Studien ist die Auswirkung der Screening-Tomosynthese auf die Intervallkarzinomrate ungeklärt [27]. Die höhere Rate Screening-detektierter invasiver Karzinome sollte mit niedrigeren Inzidenzen von invasiven Intervallkarzinomen einhergehen; andernfalls könnte eine Steigerung der Überdiagnosen vorliegen. Trotz einer 50 %igen Erhöhung der Screening-Detektionsrate mit Einsatz von DBT + SM im Vergleich zur alleinigen FFDM zeigt eine norwegische Kohortenstudie keine Unterschiede in den Raten oder histopathologischen Eigenschaften von Intervallkarzinomen [28].

TOSYMA wurde als diagnostische, randomisierte Überlegenheitsstudie im deutschen Mammographie-Screening-Programm konzipiert. Ziel ist der Vergleich der Screening-Wirksamkeitsparameter von digitaler Brust-Tomosynthese plus synthetischer 2D-Mammographie (DBT + SM) mit dem aktuellen Screening-Standard, der FFDM. Diese Multizenterstudie startete im Juni 2018 mit Studienzentrale in der Klinik für Radiologie des Universitätsklinikums Münster [29]. Der erste primäre Endpunkt der TOSYMA-Studie ist die Untersuchung der Hypothese, ob DBT + SM zu einer klinisch relevanten Erhöhung der Detektionsrate invasiver Mammakarzinome im Vergleich zur FFDM führen. Der zweite primäre Studienendpunkt ist die Prüfung kumulativer Inzidenzen invasiver Intervallkarzinome nach 24 Monaten. Sekundäre Endpunkte sind Vergleiche der Detektionsraten des duktalen Carcinoma in situ und invasiver Mammakarzinome der pT1-Kategorie, Rückrufraten, des positiven prädiktiven Werts des Rückrufs (PPV1) sowie die kumulativen 12-Monats-Inzidenzen von invasiven Intervallkarzinomen [30].

Die Rekrutierung der TOSYMA-Studie wurde zwischen Juli und Oktober 2018 in 17 Screening-Einheiten in Nordrhein-Westfalen und Niedersachsen gestartet und endet mit Abschluss der Tomosynthese-Inzidenzrunde. Studienergebnisse sind ab Herbst 2021 zu erwarten, die Erhebung der Intervallkarzinome erfordert einen Zeitraum bis 2025.

### Additiver Ultraschall bei dichter Brust

Die komplementäre Rolle der Sonographie in mammographisch dichtem Gewebe ist seit Jahrzehnten bekannt und anerkannt [16]. Allerdings ist die mögliche Rolle von Ultraschall in Screening-Programmen unklar. Weltweit gibt es kein populationsbezogenes Screening-Programm, in dem ergänzende Ultraschalluntersuchungen systematisch eingesetzt werden. Nachteile des Ultraschalls betreffen die im Vergleich zum Mammographie-Screening höheren Kontroll- und Biopsieraten, die Untersucherabhängigkeit und damit auch die effektive Qualitätssicherung.

Metaanalysen, die vorrangig auf retrospektiv erhobenen Daten mit z. T. erheblicher Studienheterogenität beruhen, bestätigen, dass die Sonographie im dichten Drüsengewebe zusätzliche Karzinome entdecken kann.

In einer aktuellen Metaanalyse [31] erhöhte die Kombination von Mammographie und Ultraschall (MX und US) bei dichtem Drüsengewebe die Sensitivität von 74 % (nur Mammographie) auf 96 %, wobei die Spezifität von 93 % auf 87 % abfiel. Wenngleich dieser Abfall gering erscheint, betrifft er bei 10.000 gescreenten Frauen pro Runde 600 Frauen oder nach 10 Runden 60 % der gescreenten Frauen.

In einer anderen Metaanalyse betrug der positive Vorhersagewert Sonographie-indizierter Biopsien lediglich < 10 % [32].

Aus Japan liegt eine randomisierte, kontrollierte Studie zur Sonographie im Screening vor, der J‑Start-Trial [33]. Hierin wurde 36.859 Frauen im Alter von 40–49 Jahren eine kombinierte MX US-Untersuchung angeboten, während 36.139 Frauen in die Kontrollgruppe (MX) eingingen. Die japanische Population, die Altersstruktur und ein jährliches Screening ist nicht mit der europäischen Screening-Population gleichzusetzen. Unter diesen spezifischen Bedingungen berichten Ohuchi et al. für MX US gegenüber MX-Screening eine Sensitivitätssteigerung von 77 % auf 91 % während die Spezifität von 91,1 % auf 87,7 % sank; die Biopsierate nahm von 1,8 % auf 4,5 % zu. Erreicht wurde eine günstigere Stadienverteilung bezüglich T2+-Karzinomen, die von 29,7 % auf 21 % sank und eine signifikante Senkung der Intervallkarzinomrate von 10 % auf 5 %.

Basierend auf den genannten Daten erschien eine Testung der Machbarkeit im MSP sinnvoll.

Es wurde die sog. DIMASOS-Studie („dichte-adaptiertes Mammographie-Sonographie-Screening“) für 30.000 teilnehmenden Frauen mit dichtem Drüsengewebe im MSP entwickelt. Frauen mit einer hohen Brustdichte (obere 15 % einer mammographisch automatisierten Dichtebestimmung) wird im Rahmen des Screenings eine ergänzende Sonographie angeboten [34]. Diese kann am selben Tag oder binnen 1 Woche nach der Screening-Mammographie durchgeführt werden. Alle teilnehmenden Ärzte erhalten eine spezielle Ausbildung und müssen den Qualitätsvorgaben (betreffend Ausbildung, Gerätetechnik, sowie Falsch-positiv-Raten) entsprechen. Im Rahmen der DIMASOS-Studie werden folgende Parameter untersucht: Zusätzliche Karzinomdetektion/1000 Frauen, zusätzlicher Recall, Biopsien, kurzfristige Kontrollen, Machbarkeit und Akzeptanz. Nach abgelaufener Rekrutierung sollen anhand einer Kontrollgruppe aus der jeweils selben Screening-Einheit an 30.000 Frauen mit dichtem Drüsengewebe (obere 15 %) Programmsensitivität, Einfluss auf die Intervallkarzinomrate und Stadienverteilung inklusive biologischer Parameter der detektierten Karzinome analysiert werden. Beginnend ab Juli 2019 wurde die DIMASOS-Studie im MSP an bis zu 20 Screening-Einheiten genehmigt. Mit einer ca. 6-monatigen Corona-bedingten Verzögerung haben im November 2020 15 Screening-Einheiten gestartet. Mit ersten Ergebnissen ist Ende 2022 zu rechnen.

### Additive Magnetresonanztomographie bei dichter Brust

Die kontrastmittelgestützte MRT der Brust ist das primäre Verfahren zur intensivierten Brustkrebs-Früherkennung bei familiärem Brust- und Eierstockkrebs [35]. Entsprechend der aktuellen Publikation des Konsortiums für familiären Brust- und Eierstockkrebs (10-Jahres-Ergebnisse anhand 14.142 Screening-Runden) wird die Programmsensitivität bei *jährlichem *Hochrisiko-Screening in Deutschland (Alter: 18,8–69,4 Jahre) mit 89,6 % angegeben. Im Programm wurden jährliche MRT-Untersuchungen und 1‑ bis 2‑jährliche Mammographien (je nach Alter und Risikoprofil) sowie je nach Risiko jährliche oder halbjährliche Sonographien durchgeführt. Unter den entdeckten Karzinomen wurden 92,9 % der Karzinome mittels MRT detektiert, wobei 30,8 % der Karzinome ausschließlich durch die MRT erkannt wurden [35].

Bislang wurde weltweit eine randomisiert-kontrollierte Studie (RCT) publiziert, die den ergänzenden Einsatz der Mamma-MRT im Screening prospektiv getestet hat, die sog. DENSE-Studie [36]. Bei dieser Studie wurde im niederländischen Mammographie-Screening-Programm die Studiengruppe mit sehr dichtem Drüsengewebe (ACR 4; 40.373 Frauen) 1:4 randomisiert und prospektiv eine ergänzende Mamma-MRT angeboten (8061 Frauen). Davon nahmen 59 % der Frauen das Angebot an und erhielten eine MRT (4783 Frauen). Die Karzinomentdeckungsrate in der Gruppe der MRT-Teilnehmerinnen lag mit 16,5/1000 deutlich über der der Kontrollgruppe (32.312 Frauen) mit einer Detektionsrate von 6,0/1000. Die Intervallkarzinomrate (eingeladen vs. nicht eingeladen) konnte von 5/1000 auf 2,5/1000 reduziert werden. Die Bedeutung der Mehrdetektion mit hohem Anteil früher, auch prognostisch günstiger Karzinome ist noch unbekannt. Die Rückrufrate zur Abklärung lag bei 9,5 %, die Biopsierate bei 6,9 % in der MRT-Gruppe, also 5‑fach bzw. 7‑fach höher als für das niederländische Screening berichtet (Angaben zur eigentlichen Kontrollgruppe sind diesbezüglich nicht publiziert).

Zwei weitere wissenschaftliche Auswertungen zur Mamma-MRT in der Brustkrebs-Früherkennung wurden kürzlich publiziert. Beide Studien untersuchten allerdings Frauen außerhalb regulärer Screening-Programme:

Kuhl et al. publizierten eine Studie, in der an 2 Instituten bei 2120 asymptomatischen Frauen mit Normalrisiko, unauffälliger Mammographie und dichtem Drüsengewebe (ACR 3 und ACR 4 gemischt) eine ergänzende Mamma-MRT (insgesamt 3861 MRT-Untersuchungen) in den Jahren von 2005 bis 2013 durchgeführt wurde. Sie berichten eine Mehrdetektion von 15,5 Karzinomen pro 1000 untersuchten Frauen (22,6/1000 in Runde 1 und 6,9/1000 für Folgerunden [37]). Der Anteil von G3-Karzinomen von 41,6 % liegt in dieser Studie weit über der erwarteten Inzidenz in der Normalpopulation und weit über der für die DENSE-Studie berichteten Rate von ca. 7 % der invasiven Karzinome und knapp 10 % aller Mammamalignome. Da diese Raten dem Vorkommen in Hochrisikokollektiven entsprechen, ist davon auszugehen, dass ein Selektionsbias vorliegt und die Studienpopulation keine durchschnittliche Screening-Population mit Normalrisiko darstellt.

Comstock et al. [38] berichten über eine Multizenterstudie, bei der an 48 Instituten bei insgesamt 1444 Frauen im Alter von 40 Jahren bis 75 Jahren mit Normalrisiko und dichtem Drüsenkörper (ACR 3 und ACR 4) eine Tomosynthese und eine zusätzliche verkürzte Mamma-MRT durchgeführt wurde. Bei dieser Studie wurde statt des üblichen MR-Protokolls mit Akquisition einer nativen und mehreren Serien nach intravenöser Kontrastmittelgabe lediglich eine native und eine Post-KM-Serie akquiriert („abbreviated MRI“). Ausgewertet wurden die Subtraktionsserien und deren 3‑D-Berechnung. Durch Weglassen der dynamischen Serien konnte die Messdauer auf unter 10 Minuten verkürzt werden. Basierend auf bislang limitierten Daten wird seitens der Autoren von Gleichwertigkeit der verkürzten Mamma-MRT mit dem Standardprotokoll ausgegangen. Mit 11,8 Karzinomen pro 1000 (detektiert mit MRT: invasive Karzinome *n* = 17, DCIS *n* = 5) vs. 4,8/1000 (detektiert mit Tomosynthese: invasive Karzinome *n* = 7, DCIS *n* = 2) zeigte die MRT eine höhere Sensitivität als die alleinige Tomosynthese bei ebenfalls höherer Spezifität der MRT von 97,4 % vs. 86,7 %. Die Ergebnisse der Tomosynthese im Screening sind bei limitierter Fallzahl allerdings sowohl für Sensitivität wie für Spezifität im internationalen Vergleich auffallend niedrig. Beispielsweise wurden in einer großen amerikanischen Screening-Studie mit primärer Tomosynthese bei dichter Brust mit ansteigenden Altersgruppen von 40–49 Jahren, 50–64 Jahren und 65–74 Jahren Detektionsraten von 5,2 bzw. 7,6 und 9,6 pro 1000 untersuchte Frauen berichtet [39].

Die Vorteile der hohen Karzinomdetektionsrate und niedrigen Intervallkarzinomrate (DENSE-Studie) müssen nachteiligen Effekten wie hohen falsch-positiven Biopsieraten sowie vermehrten Kontrollen gegenübergestellt werden. Würden bei 10 Runden pro Runde 5–7 % Biopsien und weitere ca. 8 % kurzfristige Kontrollen anfallen, würden 50–70 % dieser gescreenten Frauen nach 10 Runden mindestens eine Biopsie erlebt haben und weitere ca. 80 % eine 6‑Monats-Kontrolle.

Darüber hinaus müssen unerwünschte Wirkungen durch das Magnetfeld (durch Fremdkörper, implantierte Geräte u. a.), den i.v.-Zugang (Paravasat) und Kontrastmittelgabe (Allergie, irreversible Gadoliniumablagerungen) berücksichtigt werden.

Weitere Faktoren wie Adipositas, Klaustrophobie und Hormonsubstitution beeinflussen die Umsetzbarkeit einer MRT-Untersuchung der weiblichen Brust als Screening-Untersuchung.

Die biologische Bedeutung der Mehrdetektion ist unklar. Unter Berücksichtigung der prognostisch günstigen Malignome der DENSE- und der Comstock-Studie ist eine relevante Zahl an MRT-bedingten Überdiagnosen nicht auszuschließen. Eine weitere Herausforderung stellen histologische Klärungen von MRT-entdeckten Veränderungen mittels aufwändiger MRT-gestützter Vakuumbiopsien dar.

Aufgrund der weiterhin unzureichenden Datenlage werden sowohl die Mamma-MRT wie auch der Ultraschall aufgrund eines dichten Drüsenkörpers außerhalb einer Hochrisikokonstellation in den Europäischen Leitlinien wie in den internationalen Konsensusempfehlungen aktuell nicht als Brustkrebs-Screeningmethode empfohlen [40, 41].

## Zusammenfassung

Seit 2005 wurde in Deutschland das Mammographie-Screening-Programm etabliert, um bundesweit jeder Frau zwischen dem 50. und 69. Lebensjahr eine qualitätskontrollierte Brustkrebs-Früherkennung zu ermöglichen. Auswertungen der Leistungsparameter belegen, dass außer der Teilnahmerate bisher alle Qualitätsanforderungen dieses Programms im Kampf gegen Brustkrebs erreicht werden (z. B. Brustkrebsentdeckungsrate, Rate der Intervallkarzinome, Anteil fortgeschrittener Tumorstadien). Dementsprechend fiel auch das Feedback der Vertreter der EUREF anlässlich ihrer Visitation und Überprüfung der Nationalen Referenzzentren Mammographie aus: „Prof. Holland compliments the excellent structure of the German mammography screening programm. The programm seems to be one of the best in Europe due to its excellent quality and quality management.“

Das Mammographie-Screening-Programm führt in Deutschland zu mehr Frühdiagnosen des Mammakarzinoms als je zuvor. Frauen, die regelmäßig am Screening teilnehmen, haben – wenn dabei Brustkrebs diagnostiziert wird – signifikant seltener fortgeschrittene Tumorstadien. Diese Abnahme fortgeschrittener Brustkrebsstadien ist der relevanteste Maßstab für das Krebs-Früherkennungsprogramm, weil er mit der brustkrebsspezifischen Sterblichkeit korreliert. Zukünftig muss neben der möglichen Überdiagnostik insbesondere eine potenzielle Übertherapie differenziert betrachtet und analysiert werden.

Gesellschafts- und gesundheitspolitisch ist Krebs-Früherkennung nur in einem strukturierten und evaluierbaren Programm sinnvoll, für das eine positive Nutzen-Schaden-Bilanz auf höchstem Evidenzlevel gegeben ist [42]. Für das Mammographie-Screening-Programm liegen evidenzbasierte positive Empfehlungen vor. Die Ergebnisse belegen eine effiziente Brustkrebs-Früherkennung und deuten auf die Effektivität des Programms im Hinblick auf eine Senkung der brustkrebsspezifischen Mortalität hin. Weiterentwicklungen der Brustkrebs-Früherkennung erfordern ebenso ein hohes Evidenzlevel, erreichbar vor allem durch prospektiv-randomisierte Studien. Die enge Kooperation wissenschaftlicher Einrichtungen hat sich bewährt; begleitende Forschung unterstützt die Fortentwicklung des deutschen Screening-Programms. Neben den dargestellten laufenden Studien erscheinen insbesondere weitere Forschungsprojekte zur dedizierten Risikostratifizierung mit dem Ziel einer individualisierten Früherkennung sowie zur Optimierung des Methodeneinsatzes mit Nutzen-Risiko-Bewertung sinnvoll.

## Fazit für die Praxis

- Das Mammographie-Screening-Programm bietet allen Frauen zwischen dem 50. und 69. Lebensjahr eine qualitätskontrollierte Brustkrebs-Früherkennung.
- Nach wiederholter Screening-Teilnahme ist ein Rückgang fortgeschrittener, prognostisch ungünstiger Brustkrebsstadien nachweisbar.
- Die Reduktion der Brustkrebsmortalität durch das Mammographie-Screening-Programm wird derzeit evaluiert (ZEBra-Studie).
- Die Effektivität des Mammographie-Screening-Programms könnte durch eine höhere Teilnahmerate der eingeladenen Frauen noch weiter gesteigert werden.
- Die mittelfristig möglichen Weiterentwicklungen des Mammographie-Screening-Programms betreffen die Altersausweitung bis zum 75. Lebensjahr und den primären Einsatz der digitalen Brust-Tomosynthese (ToSyMa-Studie).
- Erweiterte Screening-Strategien für die wenigen Frauen mit extrem dichtem Drüsengewebe müssen insbesondere hinsichtlich ihrer Nutzen-Risiko-Relation wissenschaftlich überprüft werden.

## Abkürzungen

BfS: Bundesamt für Strahlenschutz
BMV‑Ä: Bundesmantelvertrag für Ärzte
DBT: Digitale Brust-Tomosynthese
DIMASOS: Dichteadaptiertes Mammographie-Sonographie-Screening
EKN: Epidemiologischen Krebsregister Niedersachsen
EUREF: European Reference Organisation for Quality Assured Breast Cancer Screening and Diagnostic Services
FFDM: Full-Field Digital Mammography
IQWiG: Institut für Qualität und Wirtschaftlichkeit im Gesundheitswesen
KBV: Kassenärztliche Bundesvereinigung
KFE-RL: Krebsfrüherkennungs-Richtlinie
KoopG: Kooperationsgemeinschaft Mammographie
LKR NRW: Landeskrebsregister Nordrhein-Westfalen
MRT: Magnetresonanztomographie
MSP: Mammographie-Screening-Programm
MX: Mammographie
RCT: Randomized Controlled Trial (randomisierte kontrollierte Studie)
RKI: Robert Koch-Institut
SM: Synthetische Mammographie
TOSYMA: Vergleich der Screening-Wirksamkeitsparameter von Digitaler Brust-Tomosynthese plus Synthetischer 2‑D-Mammographie mit Standard-2-D-FFDM
UICC: Union Internationale contre le Cancer
US: Ultraschall
